# Progress towards sustainable development goals related to sexual health

**DOI:** 10.2471/BLT.23.291163

**Published:** 2024-10-29

**Authors:** Onikepe O Owolabi, Jonathan Hopkins, Akinrinola Bankole, Jonathan Bearak

**Affiliations:** a Guttmacher Institute, New York, United States of America.; b Department of Sexual and Reproductive Health and Research, includes the UNDP/UNFPA/UNICEF/WHO/World Bank Special Programme of Research, Development and Research Training in Human Reproduction, World Health Organization, Avenue Appia 20, 1211 Geneva, Switzerland.

## Abstract

Achieving the sexual health components of sexual and reproductive health and rights as outlined in the sustainable development goals (SDGs) is integral to overall physical and mental well-being and a core part of universal health coverage. However, tracking national and global progress towards advancing the sexual health and rights of people is challenging because of the paucity of indicators to examine many of its components. To assess the state of sexual health in populations, determine service provision needs, evaluate the effectiveness of health system interventions and monitor progress in optimizing health, a comprehensive set of indicators is needed to cover every component of sexual health. Without comparable global indicators for each component of sexual health across the individual, health systems and policy levels, and disaggregated across subgroups including all genders, there is a considerable lack of insight into people’s sexual health needs and progress towards meeting those needs. This article explores the availability of global indicators for the different components of sexual health by analysing two key sources: the global indicator framework of the SDGs and the indicator index of the Global Health Observatory. We summarize the indicators for each component of sexual health using the Guttmacher–*Lancet* Commission framework, highlighting gaps in current indicators, and recommend areas where additional indicators are needed along with strategies on how to improve data availability, quality and inclusiveness.

## Résumé

Progresser sur les questions de santé sexuelle relatives à la santé et aux droits sexuels et reproductifs, qui figurent dans les objectifs de développement durable (ODD), fait partie intégrante du bien-être, tant mental que physique, et est au cœur de la couverture sanitaire universelle. Pourtant, il est difficile de mesurer les avancées réalisées dans ce domaine à l'échelle nationale et mondiale en raison du manque d'indicateurs permettant d'étudier la plupart de ses composantes. Un ensemble complet d'indicateurs est donc indispensable pour évaluer l'état de santé sexuelle des populations, identifier les besoins en prestations de services, déterminer l'efficacité des interventions des systèmes de santé et suivre les progrès visant à optimiser la santé. C'est le seul moyen d'englober toutes les composantes de la santé sexuelle. Sans indicateurs mondiaux comparables pour chaque composante de santé sexuelle au niveau des individus, des systèmes de santé et des politiques, ventilés en sous-groupes incluant tous les genres, il manque une quantité considérable d'informations concernant les besoins des gens en la matière et les progrès réalisés pour y subvenir. Le présent article se penche sur la disponibilité des indicateurs mondiaux pour les différentes composantes de santé sexuelle en analysant deux sources majeures: le cadre mondial d'indicateurs pour les ODD et l'index des indicateurs de l'Observatoire mondial de la Santé. Nous résumons les indicateurs propres à chaque composante de santé sexuelle à l'aide du cadre de la Commission Guttmacher–*Lancet*, en identifiant les lacunes présentes dans les indicateurs actuels et en signalant les domaines où des indicateurs supplémentaires sont nécessaires, tout comme des stratégies d'amélioration de la disponibilité, de la qualité et de l'inclusivité des données.

## Resumen

La consecución de los componentes de salud sexual de la salud y los derechos sexuales y reproductivos, tal como se indica en los Objetivos de Desarrollo Sostenible (ODS), es esencial para el bienestar físico y mental general y una parte fundamental de la cobertura sanitaria universal. Sin embargo, el seguimiento de los avances nacionales y mundiales hacia la promoción de la salud y los derechos sexuales de las personas es un desafío debido a la falta de indicadores para examinar muchos de sus componentes. Para evaluar el estado de la salud sexual en las poblaciones, determinar las necesidades de prestación de servicios, evaluar la eficacia de las intervenciones del sistema sanitario y supervisar los avances en la optimización de la salud, se necesita un conjunto integral de indicadores que abarque todos los componentes de la salud sexual. Sin indicadores globales comparables para cada componente de la salud sexual a nivel individual, de los sistemas sanitarios y de las políticas, y desglosados por subgrupos, incluidos todos los géneros, existe una considerable falta de conocimiento sobre las necesidades de salud sexual de las personas y los avances hacia la cobertura de esas necesidades. Este artículo explora la disponibilidad de indicadores globales para los diferentes componentes de la salud sexual mediante el análisis de dos fuentes clave: el marco de indicadores globales de los ODS y el índice de indicadores del Observatorio Global de la Salud. Se resumen los indicadores para cada componente de la salud sexual utilizando el marco de la Comisión Guttmacher-*Lancet*. Asimismo, se destacan las lagunas de los indicadores actuales y se recomiendan áreas en las que se necesitan indicadores adicionales, junto con estrategias sobre cómo mejorar la disponibilidad, la calidad y la inclusividad de los datos.

## ملخص

إن تحقيق مكونات الصحة الجنسية، والصحة الإنجابية، والحقوق الجنسية كما هو موضح في أهداف التنمية المستدامة (SDG)، يشكل جزءًا لا يتجزأ من الرفاهية البدنية والعقلية العامة، وجزءًا جوهريًا من التغطية الصحية الشاملة. ومع ذلك، فإن تتبع التقدم الوطني والعالمي نحو الارتقاء بالصحة الجنسية وحقوق الأشخاص، هو بمثابة تحدي بسبب ندرة المؤشرات لفحص العديد من مكوناتها. لتقييم حالة الصحة الجنسية لدى السكان، وتحديد احتياجات تقديم الخدمات، وتقييم فعالية تدخلات النظام الصحي، ورصد التقدم في تحسين الصحة، فإن هناك حاجة إلى مجموعة شاملة من المؤشرات لتغطية كل مكون من مكونات الصحة الجنسية. ودون وجود مؤشرات عالمية قابلة للمقارنة لكل مكون من مكونات الصحة الجنسية لدى الفرد، والأنظمة الصحية، ومستويات السياسة، وتفكيكها عبر الفئات الفرعية بما يشمل كلا الجنسين، فإن هناك نقص ملموس في رؤية الاحتياجات الجنسية للأشخاص، والتقدم نحو تلبية هذه الاحتياجات. تستكشف هذه المقالة مدى توفر المؤشرات العالمية للمكونات المختلفة للصحة الجنسية من خلال تحليل مصدرين رئيسيين: إطار عمل المؤشرات العالمية لأهداف التنمية المستدامة، وفهرس مؤشرات مرصد الصحة العالمية. ونلخص مؤشرات كل مكون من مكونات الصحة الجنسية باستخدام إطار عمل لجنة Guttmacher– *Lancet *، مع تسليط الضوء على الفجوات في المؤشرات الحالية، والتوصية بالمجالات التي تحتاج إلى مؤشرات إضافية، إلى جانب استراتيجيات حول كيفية تحسين توافر البيانات، وجودتها، وشمولها.

## 摘要

可持续发展目标 (SDG) 指出要确保人们享有性与生殖健康以及相关权利，而实现其中的性健康组成部分，是追求身心整体健康不可或缺的部分，也是全民健康覆盖的核心部分。然而，由于缺乏用来考察性健康许多组成部分的指标，在国内和全球范围内跟踪在确保人们享有性健康和相关权利方面所取得的进展具有一定的挑战性。为了评估人口的性健康状况、确定服务提供需求、评估卫生系统干预措施的有效性，并监测在优化健康方面所取得的进展，需要制定一套涵盖性健康各个组成部分的全面性指标。在个人、卫生系统和政策层面上，性健康的每个组成部分均无类似的全球指标，也不存在包括所有性别在内的子群体分类指标，因此很难深入了解人们的性健康需求和在满足这些需求方面所取得的进展。本文通过分析两个重要来源探讨了使用全球指标评估性健康不同组成部分的可行性，即：SDG 的全球指标框架和全球卫生观察站 (Global Health Observatory) 的指标指数。我们借助 Guttmacher-*Lancet* 委员会的框架总结了适用于性健康各个组成部分的指标，重点说明了现有指标存在的缺陷，并就需要增加指标的领域以及制定提高数据的可用性、质量和包容性相关战略给出了建议。

## Резюме

Укрепление компонентов сексуального здоровья в рамках сексуального и репродуктивного здоровья и прав, как указано в Целях в области устойчивого развития (ЦУР), является неотъемлемой частью общего физического и психического благополучия и одной из основных составляющих всеобщего охвата услугами здравоохранения. Однако отслеживание прогресса на национальном и глобальном уровнях в деле укрепления сексуального здоровья и прав людей затруднено из-за нехватки показателей для изучения многих его компонентов. Для оценки состояния сексуального здоровья населения, определения потребностей в услугах, оценки эффективности вмешательств в систему здравоохранения и мониторинга прогресса в оптимизации здоровья необходим комплексный набор показателей, охватывающий все компоненты сексуального здоровья. В отсутствие сопоставимых глобальных показателей по каждому компоненту сексуального здоровья на уровне отдельных людей, систем здравоохранения и политики, а также в разбивке по подгруппам, включая представителей всех полов, наблюдается значительный недостаток информации о потребностях людей в том, что касается сексуального здоровья, и прогрессе в удовлетворении этих потребностей. В этой статье рассматривается наличие глобальных показателей для различных компонентов сексуального здоровья на основе анализа двух ключевых источников: глобальной системы показателей ЦУР и индекса показателей Глобальной обсерватории здравоохранения. В статье обобщены показатели по каждому компоненту сексуального здоровья с использованием системы Комиссии Гутмахера-*Ланцета*, выделены пробелы в существующих показателях и рекомендованы области, в которых необходимы дополнительные показатели, а также стратегии по улучшению доступности, качества и инклюзивности данных.

## Introduction

In the past 30 years since the International Conference on Population and Development, the international development community has increasingly acknowledged that individual health, well-being and economic growth depend on sexual and reproductive health and rights.^1^^–^^3^ Since the conference, there has been great attention towards reproductive health and reproductive rights.^4^^,^^5^ However, despite the inextricable link to reproductive health and rights, sexual health and rights have remained narrowly construed, politicized and contentious.^6^ As a result, global and national conversations around funding, service planning, integration, service provision and monitoring tend to sidestep more expansive definitions of sexual and reproductive health, focusing instead on reproductive, maternal, newborn, child and adolescent health. This focus can be seen in the categorization of the top 100 health indicators published in 2018.^7^ The lack of an internationally agreed definition of sexual health has led to limited consensus across countries and stakeholders in the international development community regarding its scope. Even where broad principles are agreed upon, reaching consensus on the specifics remains challenging.

The World Health Organization’s (WHO) working definition of sexual health is “a state of physical, emotional, mental and social well-being in relation to sexuality.”^8^ Sexual health and well-being include safe and pleasurable sexual experiences free from coercion, discrimination, abuse and violence, in addition to freedom from diseases such as sexually transmitted infections.^9^ However, the international development community has yet to reach a consensus on a core set of indicators to evaluate access to comprehensive sexual health services within the health-care system, and the impact of sexual health on people’s overall health and lives. Consequently, while some stakeholders present clear visions of sexual health, disease-specific components of sexual health, such as sexually transmitted infections and human immunodeficiency virus (HIV) infection in particular, dominate the agreed-upon indicators.

Clear indicators for sexual health and well-being are essential for effectively advocating for the delivery of comprehensive sexual health services within universal health coverage (UHC), and for monitoring progress in the achievement of sexual health at the population level. In this paper, we examine the availability and adequacy of global indicators used to understand the current state of sexual health and track progress in population-level health outcomes over time. While we do not assess the extent, quality or recency of the data for these indicators, nor identify in which countries they are currently collected, we provide an initial attempt to compile the sexual health and well-being indicators.

## Components of sexual health

In 2018, the Guttmacher–*Lancet* Commission proposed a new definition of sexual and reproductive health and rights that articulated the breadth of issues and services included. The seminal report outlined nine interventions necessary to guarantee comprehensive sexual and reproductive health services.^9^ As sexual health and reproductive health are deeply entwined,^8^ very few, if any, indicators exclusively apply to either sexual or reproductive health. Nevertheless, as the basis of our indicator review and selection, we roughly categorized these nine interventions into five components primarily related to sexual health, and four primarily related to reproductive health (Table 1). Subsequently, to ascertain whether the full breadth of sexual health is being measured, we categorized the sexual health indicators identified and reviewed into the five sexual health components: (i) sexually transmitted infections, including HIV; (ii) cancers of the reproductive system; (iii) gender-based violence, including violence against women; (iv) comprehensive sexuality education; and (v) sexual health and well-being (Table 1).

**Table 1:** The components of sexual health and reproductive health

Component	Definition and scope
**Sexual health**	
Sexually transmitted infections, including HIV	Prevention, detection and treatment of sexually transmitted infections, including HIV and reproductive tract infections
Cancers of the reproductive system	Prevention, detection and treatment of reproductive cancers, especially cervical cancer
Gender-based violence, including violence against women	Prevention, detection and management of sexual and gender-based violence and coercion
Comprehensive sexuality education	Accurate information and counselling on sexual and reproductive health, including evidence-based, comprehensive sexuality education
Sexual health and well-being	Information, counselling and care related to sexual function and satisfaction
**Reproductive health**	
Contraception	A choice of safe and effective contraceptive methods
Maternal and newborn health	Safe and effective antenatal, childbirth and postnatal care
Safe abortion	Safe and effective abortion services and care
Infertility	Prevention, management and treatment of infertility

HIV: human immunodeficiency virus.

Note: we based the components on the nine interventions outlined in *Accelerate progress – sexual and reproductive health and rights for all: report of the Guttmacher–Lancet Commission*.^9^

## Current key indicators

To explore how the international development community currently measures sexual health globally, we reviewed the indicators found in the *Global indicator framework for the sustainable development goals and targets of the 2030 agenda for sustainable development*^10^ and the indicators index of the WHO Global Health Observatory.^11^ We chose the sustainable development goal (SDG) framework because the SDGs include universal access to sexual and reproductive health-care services, with targets broadly related to sexual and reproductive health and rights. We chose the Global Health Observatory repository because it is the hub of the WHO statistical information system, aggregating reliable and comparable health indicators from WHO Member States. The repository contains reports, country statistics, a map gallery and standardized indicator registry.^12^ In total, the SDG framework includes 244 indicators,^10^ while the Global Health Observatory index contains 1578 indicators.^11^ Two authors independently extracted indicators related to sexual health, and two other authors reviewed the choice of extracted indicators and chosen sexual health component categorization. We included indicators if they directly relate to sexual health or if they measure a health system, legal or policy response to a sexual health component. We defined indicators directly related to sexual health as those indicators measuring the incidence, prevalence, prevention or treatment of a sexual health component. For example, for indicators related to hepatitis, we included the incidence or prevalence of hepatitis B and C, as they are sexually transmitted, but did not include the incidence of chronic hepatitis, which is not sexually transmitted. Subsequently, we categorized all the eligible indicators into the five sexual health components. Only one indicator (legal environment) was classified into two of the five sexual health components – comprehensive sexuality education and sexual health. Indicators that primarily measured reproductive health outcomes, such as adolescent birth rate, were not included despite linkages to sexual health and well-being.^13^ A range of other indicators that are affected by, but which do not directly measure sexual health outcomes, were also not included, such as age-standardized suicide rates.

We considered a total of 73 sexual health indicators, to be primarily indicators of sexual health and well-being (Table 2). Ten indicators from the SDG framework and 63 indicators from the Global Health Observatory. The number of indicators pertaining to each of the sexual health components are outlined in Table 3.

**Table 2:** Categorization of sexual health indicators identified from the indicator sources of the SDGs^10^ and the Global Health Observatory^11^

Component, source	Indicator^a^	Type
**Sexually transmitted infections including HIV**		
SDG	Number of new HIV infections per 1 000 uninfected population, by sex, age and key population (3.3.1)	Incidence
GHO	HIV – Estimated antiretroviral therapy coverage among children	Treatment
GHO	HIV – Estimated antiretroviral therapy coverage among people living with HIV (%)	Treatment
GHO	HIV – Estimated number of children living with HIV	Prevalence
GHO	HIV – Estimated number of pregnant women living with HIV	Prevalence
GHO	HIV – Estimated percentage of pregnant women living with HIV who received antiretrovirals for preventing mother-to-child transmission	Treatment
GHO	HIV – New HIV infections (per 1 000 uninfected population)	Incidence
GHO	HIV – New HIV infections among adults 15–24 years old (per 1 000 uninfected population)	Incidence
GHO	HIV – Number of new HIV infections	Incidence
GHO	HIV – Number of people (all ages) living with HIV	Prevalence
GHO	HIV – Number of people dying from HIV-related causes	Deaths
GHO	HIV – Number of pregnant women living with HIV who received antiretrovirals for preventing mother-to-child transmission	Treatment
GHO	HIV – People who received HIV testing and counselling	Testing
GHO	HIV – Prevalence of HIV among adults aged 15 to 49 years (%)	Prevalence
GHO	HIV – Reported number of children receiving antiretroviral therapy	Treatment
GHO	HIV – Reported number of people receiving antiretroviral therapy	Treatment
GHO	HIV – Reported number of people receiving antiretroviral therapy, month and year of report	Treatment
GHO	HIV – testing and counselling facilities	Health system
GHO	Testing and counselling facilities, estimated number per 100 000 adult population	Health system
GHO	Testing and counselling facilities, reported number	Health system
GHO	Testing and counselling facilities, reporting period	Health system
GHO	Population aged 15–24 years with comprehensive correct knowledge of HIV/AIDS (%)	Prevention
GHO	Prevalence of condom use by adults during higher-risk sex (15–49 years) (%)	Prevention
GHO	Women accessing antenatal care (ANC) services who were tested for syphilis (%), reported	Testing
GHO	Antenatal care attendees positive for syphilis who received treatment (%), reported	Treatment
GHO	Antenatal care attendees who were positive for syphilis (%), reported	Prevalence
GHO	HIV-positive TB patients on ART (antiretroviral therapy) (%)	Treatment
GHO	TB patients with known HIV status (%)	Testing
GHO	Tested TB patients HIV-positive (%)	Prevalence
GHO	Treatment success rate: HIV-positive TB cases	Treatment
GHO	Hepatitis - new infections	Incidence
GHO	Hepatitis - number of chronic hepatitis B-infected persons treated	Treatment
GHO	Hepatitis - number of persons initiated hepatitis C treatment, latest year and cumulative over a period of years	Treatment
GHO	Hepatitis- diagnosis coverage of chronic hepatitis (HBV and HCV) as a percentage of infected	Prevalence
GHO	STI: Incident cases of chlamydia in 15–49 year olds (in thousands)	Incidence
GHO	STI: Incident cases of chlamydia in 15–49 year olds (per 1 000)	Incidence
GHO	STI: Incident cases of gonorrhoea in 15–49 year olds (in thousands)	Incidence
GHO	STI: Incident cases of gonorrhoea in 15–49 year olds (per 1 000)	Incidence
GHO	STI: Incident cases of syphilis in 15–49 year olds (in thousands)	Incidence
GHO	STI: Incident cases of trichomoniasis in 15–49 year olds (in thousands)	Incidence
GHO	STI: Incident cases of trichomoniasis in 15–49 year olds (per 1 000)	Incidence
GHO	STI: Incident rate of active syphilis (per 1 000)	Incidence
GHO	STI: Prevalence of active syphilis in 15–49 year olds (%)	Prevalence
GHO	STI: Prevalence of chlamydia in15–49 year olds (%)	Prevalence
GHO	STI: Prevalence of gonorrhoea in 15–49 year olds (%)	Prevalence
GHO	STI: Prevalence of trichomoniasis in 15–49 year olds (%)	Prevalence
GHO	STI: Prevalent cases of active syphilis (in thousands)	Prevalence
GHO	STI: Prevalent cases of chlamydia in 15–49 year olds (in thousands)	Prevalence
GHO	STI: Prevalent cases of gonorrhoea in 15–49 year olds (in thousands)	Prevalence
GHO	STI: Prevalent cases of trichomoniasis in 15–49 year olds (in thousands)	Prevalence
**Cancers of the reproductive system**		
GHO	HPV immunization coverage estimates among primary target cohort (9–14 years old girls) (%)	Prevention
GHO	Prevalence of cervical cancer screening among women aged 30–49 years (%)	Testing
GHO	Most widely used screening method in national cervical cancer screening programme	Health system
GHO	Type of national cervical cancer screening programme	Health system
GHO	Existence of national screening programme for breast cancer	Testing
**Gender-based violence including violence against women**		
SDG	Proportion of ever-partnered women and girls aged 15 years and older subjected to physical, sexual or psychological violence by a current or former intimate partner in the previous 12 months, by form of violence and by age (5.2.1)	Incidence
SDG	Proportion of women and girls aged 15 years and older subjected to sexual violence by persons other than an intimate partner in the previous 12 months, by age and place of occurrence (5.2.2)	Incidence
SDG	Proportion of persons victim of physical or sexual harassment, by sex, age, disability status and place of occurrence, in the previous 12 months (11.7.2)	Incidence
SDG	Proportion of population subjected to (a) physical violence, (b) psychological violence and (c) sexual violence in the previous 12 months (16.1.3)	Incidence
SDG	Proportion of young women and men aged 18–29 years who experienced sexual violence by age 18 (16.2.3)	Prevalence
GHO	Intimate partner violence prevalence among ever-partnered women in the previous 12 months (%)	Incidence
GHO	Intimate partner violence prevalence among ever-partnered women in their lifetime (%)	Prevalence
GHO	Intimate partner violence: Extent of implementation of dating violence prevention programmes	Prevention
GHO	Lifetime prevalence of child sexual abuse (%)	Prevalence
GHO	Non-partner sexual violence prevalence (%)	Prevalence
GHO	Proportion of ever-partnered women and girls aged 15–49 years subjected to physical and/or sexual violence by a current or former intimate partner in the previous 12 months	Incidence
**Comprehensive sexuality education**		
SDG	Number of countries with laws and regulations that guarantee full and equal access to women and men aged 15 years and older to sexual and reproductive health care, information and education (5.6.2)	Laws and policies
**Sexual health and well-being**		
SDG	Proportion of women aged 20–24 years who were married or in a union before age 15 and before age 18 (5.3.1)	Prevalence
SDG	Proportion of girls and women aged 15–49 years who have undergone female genital mutilation, by age (5.3.2)	Prevalence
SDG	Proportion of women aged 15–49 years who make their own informed decisions regarding sexual relations, contraceptive use and reproductive health care (5.6.1)	Well-being
SDG	Number of countries with laws and regulations that guarantee full and equal access to women and men aged 15 years and older to sexual and reproductive health care, information and education (5.6.2)	Laws and policies
GHO	Proportion of women aged 20–24 years who were married or in a union by age 15 (%)	Prevalence
GHO	Proportion of women aged 20–24 years who were married or in a union by age 18 (%)	Prevalence
GHO	Proportion of girls and women aged 15–49 years who have undergone female genital mutilation/cutting (%)	Prevalence

AIDS: acquired immunodeficiency syndrome; GHO: Global Health Observatory; HBV: hepatitis B virus; HCV: hepatitis C virus; HIV: human immunodeficiency virus; HPV: human papillomavirus; SDG: sustainable development goals; STI: sexually transmitted infection.

^a^ Text as given in source. For SDG indicators, the numbers in the parentheses are the indicator numbers.

Note: we based the components on the nine interventions outlined in *Accelerate progress – sexual and reproductive health and rights for all: report of the Guttmacher–Lancet Commission*.^9^

**Table 3:** Number of indicators for each sexual health component in the indicator sources of the SDGs^10^ and the Global Health Observatory^11^

Sexual health component	No. of indicators	
	SDGs	GHO
Sexually transmitted infections including HIV	1	49
Cancers of the reproductive system	0	5
Gender-based violence including violence against women	5	6
Comprehensive sexuality education	1	0
Sexual health and well-being	4	3
**Total**	**10^a^**	**63**

GHO: Global Health Observatory;HIV: human immunodeficiency virus; SDGs: sustainable development goals.

^a^ The total does not sum to 11 because one indicator is included under both comprehensive sexuality education and sexual health and well-being.

Note: we based the components on the nine interventions outlined in *Accelerate progress – sexual and reproductive health and rights for all: report of the Guttmacher–Lancet Commission*.^9^

Of all the sexual health components, 50 (68%) indicators relate to sexually transmitted infections. Among these, HIV indicators are the most prevalent, accounting for 25 (51%) of the 49 sexually transmitted infection indicators in the Global Health Observatory, and the only SDG indicator. Other sexually transmitted infection indicators cover syphilis, chlamydia, gonorrhoea, trichomoniasis and hepatitis B and C. One indicator measures the prevention of sexually transmitted infections.

In the SDG framework, the largest number of sexual health indicators (five out of 10; 50%) focus on gender-based violence. Similarly, in the Global Health Observatory, gender-based violence indicators are the second most common, with six out of 63 (10%), following those related to sexually transmitted infections. These indicators cover physical, sexual and psychological violence and harassment, child sexual violence and the prevention of dating violence. While most of these indicators focus on women and girls, three of the SDG indicators are broader, looking at aspects of gender-based violence among women and men.

We found five (7%) indicators for reproductive system cancers, all included in the Global Health Observatory. These focus on cervical cancer, human papilloma virus (HPV) immunization and breast cancer screening.

We identified seven (10%) sexual health and well-being indicators (four SDG indicators and three from the Global Health Observatory). They include child marriage; female genital mutilation and/or cutting; the ability of women to make informed decisions regarding sexual relations, contraceptive use and reproductive health care; and laws and regulations that guarantee access to sexual health care. Child marriage relates to both reproductive and sexual health. Child marriage increases the chance of adolescent pregnancy, gender-based violence and HIV, and reduces the possibility of negotiating consensual sex, which reduces overall sexual health and well-being.

One SDG indicator and none of the Global Health Observatory indicators address comprehensive sexuality education, with the SDG indicator measuring if laws and policies guarantee access to such education.

Of the 73 indicators of sexual health and well-being identified, 43 (59%) focus on quantifying the population prevalence or incidence of a condition. Additionally, 17 (23%) measure aspects of health service uptake, such as the proportion of people tested, screened or treated. Only one indicator measures adverse outcomes, specifically, HIV-related deaths. Five (7%) indicators measure prevention activities such as knowledge of HIV, HPV vaccination, or dating violence prevention programmes. Six (8%) indicators measure health service availability, such as the number of HIV testing sites or having a cervical cancer screening programme. Finally, one indicator measures the policy environment and one measures individual agency (Table 2).

Most sexual health indicators from these sources relate to sexually transmitted infections and gender-based violence, primarily focusing on the prevalence or incidence of disease or infirmity. Notably, comprehensive sexuality education is not measured at the individual level, with no data on the proportion of young people who have sexual health knowledge or access to such information. Sexual health has an important well-being element that applies to people regardless of their sexual activity or partnership status.^6^ Yet, none of the Global Health Observatory indicators and just one SDG indicator relates to sexual well-being: the proportion of women who make their own informed decisions regarding sexual relations, contraceptive use, and reproductive health care (SDG 5.6.1). Similarly, there are no indicators measuring menstrual health and hygiene or gynaecologic or urologic dysfunction.

## Looking ahead

The current sexual health indicators are inadequate as they primarily focus on the prevalence of HIV, sexually transmitted infections and gender-based violence, while overlooking other important domains of sexual health. Investments are needed to identify or develop a comprehensive set of harmonized indicators that effectively monitor access to and quality of all the domains of sexual health within national systems. Additionally, it is important to identify both available and new approaches for collecting these data across multiple sectors in different countries. The continued failure to measure sexual health indicators hinders a clear understanding of people’s experiences and limits opportunities to strengthen health systems’ capacity to meet their needs. Additionally, despite the importance of education and information for awareness of sexual health needs and access to services, comprehensive sexuality education is almost absent among the available indicators. Therefore, efforts to develop indicators should aim to achieve three objectives. 

First, we need to develop and include measures of the positive aspects of sexual health, for example, pleasure, healthy communication, relationship quality and quality of life.^14^ Sexual health and well-being are fundamental to human sexuality and social interactions. Hence, indicators should not only focus on preventing and treating adverse sexual outcomes but also on improving the quality of sexual experiences and relationships. Additional areas that could be included are dysmenorrhea, erectile dysfunction, dyspareunia and menopause, all of which directly affect the sexual experiences.^15^ As others have already called for, “sexual violence, pleasure and satisfaction should be routinely incorporated in sexual health datasets, as both explanatory variables and outcomes in studies of sexual behaviour, and as endpoints in trials of the effectiveness of sexual health interventions.”^16^

Second, we need to develop and include more indicators that measure the larger environment in which sexual health care is provided, integrating these into routine national and global monitoring. Such indicators include existence of national and subnational laws and policies, social norms and attitudes, and access to sexual health information and education.^9^ Although accurate information is important for the awareness of sexual health needs and access to services, the number of indicators routinely collected is currently unequal between the five components of sexual health. New measures should focus on not only the type of education and information available but also on the sources of information. This approach is particularly important in today’s digital environment, where unverified information is increasingly accessible through social media. For adolescents and young people, knowledge gained through school-based comprehensive sexuality education and from trained providers is preferable.^9^

Third, sexual health questions should be integrated into broader health data collection tools to provide a more holistic understanding of the factors affecting sexual health, such as mental health or noncommunicable diseases. For instance, people with noncommunicable diseases have an elevated risk of sexual dysfunction.^17^ Improved data on sexual health will enable providers, researchers and policy-makers to better understand what factors drive sexual health and well-being.

In addition to developing new indicators, organizations like WHO should agree on a core set of sexual health and well-being indicators to be prioritized for inclusion in global reporting. WHO should also propose a compendium of additional sexual health indicators for governments to choose from, and offer support for standalone in-depth studies that aim to answer contextually relevant questions. This approach can include both strengthening data collection modules in health management information systems, and conducting surveys and other smaller studies to test our new indicators in diverse population groups. Other potential sources of data may include regular surveillance, and surveys using digital tools and applications to capture broader measures of sexual health and well-being that are usually not included in health management information system data. A mix of approaches is important because regular surveillance, for example in health and demographic surveillance sites or other cohort studies, can help capture the effects of changes in technology, policies and sociocultural contexts of sexuality.^18^ Furthermore, population-based surveys can capture emerging aspects of sexual health including group norms and individual attitudes, knowledge, preferences, health perspectives, experiences and behaviours that traditional facility-based information systems cannot. These surveys can also measure relationship satisfaction and other positive aspects of sexuality representing the population of interest. Such data can provide insights into diverse and evolving aspects of sexual health, which can be shared with communities, used to develop advocacy messages and inform policy and community health priorities.^18^

The international development community lacks consensus on the definitions of some sexual health terms. For instance, some people use the term sexual health, while others prefer healthy sexuality,^19^ yet these two terms may have different meanings. Some argue that healthy sexuality could be misused to label certain forms of sexuality, such as same-sex relationships, as sexually unhealthy in contexts where they are considered unacceptable.^20^ Researchers in sexual and reproductive health and rights need to propose and test new measures for neglected components of sexual health that go beyond a binary gender approach, accommodating people of all genders. To achieve a common understanding and consistent application of sexual health terms and questions used to produce indicators, cross-country studies and comparisons are needed, along with collaboration with national agencies and multilateral organizations that collate data. Agreeing on key concepts related to sexual health and their definition is needed to prevent stigmatization of certain sexual behaviours, reduce discrimination in access to services and ensure comparability across different contexts.

While sexual health is important across the life course, current data disproportionately focuses on women and girls in their reproductive years, with the notable exception of HIV data. Therefore, data collected must include key sociodemographic and contextual characteristics to provide a more comprehensive understanding of sexual health needs and if these needs are currently being met. For example, data disaggregation should include people of all age groups; diverse sexual orientation and gender identities; key populations such as sex workers and people who inject drugs; people with disabilities; and other vulnerable and minority groups, as relevant to the country context. Health service data should include the level of health service provider (community, clinic, health centre or hospital) and location (rural, urban or peri-urban as relevant within the context). 

The tendency to limit the conceptualization of sexual health to people of reproductive age ignores the well-being of a considerable proportion of the population. Of particular importance is measuring sexual health among children and adolescents, especially in contexts where child marriage is common and where social norms dictate the sexuality of young people. Data collection efforts must also address men’s needs and experiences. Currently, men’s involvement in sexual and reproductive health and rights interventions primarily focus on addressing gender power dynamics that favour men and reinforce harmful gender norms. While essential to achieve gender equality, the need to focus on men’s sexual health is equally critical. As noted by others, “there remains a gap in evidence and practice around better engaging men as [sexual and reproductive health] clients and service users in their own right, including providing high-quality and accessible male-friendly services.”^21^ Furthermore, research, programme and policy actions are required to better engage men in improving their sexual health, which can also improve the sexual health of their partners.^22^

Our analysis has some limitations. First, we only assessed indicators listed in SDG and Global Health Observatory sources. Other databases, such as the Joint United Nations Programme on HIV/AIDS (UNAIDS) AIDSinfo, also include sexual health indicators. Further, a range of sexual health data are collected at national and sub-national levels, for example through demographic health surveys, and may be reported elsewhere.^23^ A more comprehensive review of all sexual health indicators could support the development of a global compendium of sexual health indicators. Furthermore, we have not assessed which indicators are actually routinely collected and reported over time nor how these data are disaggregated. Additional research should be conducted to assess data availability, comparability and coverage for various sexual health indicators, since data availability may unexpectedly be higher for low- and middle-income countries than high-income countries.^24^

## Conclusion

Sexual health is a fundamental part of overall health and well-being. Current indicators of sexual health and well-being in two of the largest sources for global health indicators mainly measure the prevalence and incidence of disease, and monitoring health system responses to avert morbidity and mortality. The lack of indicators related to the legal or policy environment, comprehensive sexuality education or sexual health and well-being, reinforce a narrow view of sexual health. As a result, current indicators fail to capture the experience of sexual health, the impacts of social structures and norms on bodily autonomy, and the importance of sexual health and well-being to overall quality of life.

Measuring all components of sexual health has been challenging because of insufficient political will and lack of relevant data.^14^ The international community must reconsider data collection priorities to create an ecosystem with positive health and well-being indicators across all five components of sexual health. Indicators should be conceptualized to reflect human rights within which people experience their sexual health, both during and after the reproductive years. This approach will make the sexual health components of sexual and reproductive health and rights more visible and will be much harder to overlook, ultimately supporting efforts to achieve UHC.
